# Pilot Feasibility Study of an Anti-Stigma Intervention for Romanian Psychiatry Trainees

**DOI:** 10.3390/healthcare14131972

**Published:** 2026-07-02

**Authors:** Elena Andreea Manescu, Claire Henderson, Adriana Mihai

**Affiliations:** 1 Department of Psychiatry, George Emil Palade University of Medicine, Pharmacy, Science, and Technology of Targu Mures, 540142 Targu-Mures, Romania; 2 Public Mental Health, Health Services and Population Research Department, David Goldberg Centre, King’s College London Institute of Psychiatry, Psychology and Neuroscience, De Crespigny Park, London SE5 8AF, UK

**Keywords:** mental health stigma, anti-stigma intervention, mental health professionals

## Abstract

**Background:** Stigma toward people with mental illness remains a major barrier to quality care, persisting even among mental health professionals. This study evaluated the effectiveness and feasibility of a structured anti-stigma intervention for psychiatry trainees. **Methods:** Outcomes were assessed at baseline, post-intervention, and 3-month follow-up using a knowledge quiz, the Attitudes to Addressing Stigma and Discrimination Scale (ASTAD), and an Objective Structured Clinical Examination (OSCE). Feasibility outcomes were also evaluated. **Results:** Significant improvements were observed across all measures immediately after training, with medium to large effect sizes. Knowledge scores increased post-intervention (+6.68; 95% CI 5.86–7.50) and remained significantly higher at follow-up (+6; 95% CI 4.99–7.01). Total ASTAD scores improved post-intervention (+5; 95% CI 3.26–6.74) but did not maintain at follow-up. OSCE scores increased by 0.95 points (95% CI 0.62–1.28) post-training. The intervention reached 40% of eligible trainees, with high satisfaction and perceived relevance to clinical practice. Participants reported increased awareness of stigma, improved communication skills, and greater confidence in addressing stigma in patient interactions. Experiential learning methods, including role-play and case discussions, were identified as the most impactful components. Barriers to participation included workload, scheduling constraints, and limited institutional support. Participants recommended integrating the intervention into formal training curricula. **Conclusions:** The intervention was feasible, acceptable, and effective in improving stigma-related knowledge, attitudes, and clinical skills among psychiatry trainees. While knowledge gains were sustained, attitudinal and behavioral changes were only partially maintained, highlighting the need for longitudinal integration and ongoing reinforcement.

## 1. Introduction

While public stigma has been the focus of numerous campaigns and interventions [1,2,3,4,5,6], the recognition of stigma within healthcare, and particularly within mental healthcare, along with the development of evidence-based strategies to address it, remains in its early stages [7,8,9,10,11,12,13,14,15,16]. Research indicates that even clinicians with extensive mental health knowledge may hold stigmatizing attitudes and engage in discriminatory practices, including paternalism, assumptions of incurability, prioritizing symptom control over personal recovery, therapeutic pessimism, labeling, moral judgment, and diminished empathy [7,17,18,19,20]. Among healthcare providers, including psychiatrists, stigmatizing attitudes have been associated with reduced patient engagement, lower adherence to treatment, and diminished trust in clinicians [7,20,21,22]. Such attitudes can undermine the therapeutic alliance, a cornerstone of effective psychiatric care, by fostering feelings of judgment or rejection. As a result, care quality suffers, and patients are less likely to maintain engagement or achieve lasting recovery.

With appropriate training, however, mental health professionals have the potential to act as key agents in combating stigma at multiple levels. Their professional expertise and direct engagement with individuals affected by mental illness position them to influence public attitudes, organizational culture, and advocate for structural and policy reforms. Accordingly, they represent a priority group for anti-stigma and health advocacy interventions [23]. Understanding the mechanisms through which professionals’ attitudes and systemic factors perpetuate stigma, and developing evidence-based, contextually tailored interventions to address them, is therefore essential for promoting effective mental health care.

A recent review on the effectiveness of stigma reduction and health advocacy interventions highlights several key findings [24]. First, there is limited evidence regarding the feasibility and effectiveness of interventions targeting mental health professionals, with most available studies originating from high-income countries. Second, interventions should be contextually tailored; that is, developed in alignment with the specific roles of mental health professionals and informed by a prior situational analysis. Third, the content of such interventions should emphasize patient empowerment, a goal that should also be achieved by involving individuals with lived experience in program delivery. Fourth, interactive and experiential methods have demonstrated superior effectiveness compared to didactic approaches. Finally, the evaluation should focus on behavioral change.

Given that the majority of existing studies have been conducted in high-income countries (HICs) [9,14,15,25,26], generalizing their findings to low- and middle-income countries (LMICs) presents considerable challenges due to differences in health systems, workforce capacity, and professional roles, which may shape both stigma manifestations and the feasibility and effectiveness of anti-stigma interventions.

Although Romania has recently been classified as an HIC, it continues to present LMIC features. Its mental health system faces persistent structural and operational deficits. Funding remains well below European Union standards [27], contributing to a low density of mental health professionals. Consequently, practitioners are required to assume duties beyond their formal job descriptions, factors that drive burnout and reinforce stigma. Additionally, the underdevelopment of community-based services, coupled with inefficient coordination between mental health institutions, partially due to the absence of a cohesive conceptual framework, further reflects critical structural issues [28]. Compounding these systemic barriers, formal education on mental health stigma is absent from undergraduate medical curricula and psychiatric residency training [29], despite evidence of limited stigma-related knowledge and stigmatizing attitudes and behaviors among health and mental health professionals [19] and widespread public stigma in Romania [28].

Although increasing interest in anti-stigma interventions in healthcare, several important gaps remain in the existing literature. First, evidence regarding interventions targeting mental health professionals, and psychiatry trainees specifically, remains limited compared with interventions directed at medical students or the general public. Second, previous interventions have frequently focused on attitudinal outcomes, while fewer studies have incorporated behavioral components, health advocacy principles, or feasibility outcomes relevant for implementation in routine training settings.

An additional and often underexplored mechanism relevant to intervention design concerns causal beliefs about mental illness. In particular, biogenetic or medical-model causal beliefs conceptualize mental health problems primarily as manifestations of underlying biological pathology. Although such explanations may reduce personal blame and reinforce perceptions that symptoms are not under voluntary control, evidence suggests that they may simultaneously reinforce other stigmatizing beliefs, including assumptions of dangerousness, unpredictability, chronicity, and increased social distance [30,31].

These mixed effects indicate that causal attribution alone is insufficient to reduce stigma and may, depending on how it is framed and integrated into clinical understanding, contribute to the persistence of stigmatizing attitudes within healthcare settings.

Addressing these needs, and following evidence-based recommendations, we implemented a pilot intervention for mental health professionals, informed by a situational analysis [19] and grounded in the framework of responding to experienced and anticipated discrimination [23]. In this framework, experienced discrimination refers to actual episodes of unfair treatment, exclusion, restricted opportunities, or differential treatment encountered because of mental health difficulties or psychiatric diagnosis, whereas anticipated discrimination refers to the expectation of future rejection, devaluation, or discriminatory treatment, which may lead individuals to conceal difficulties, avoid help-seeking, or restrict social and occupational participation [32].

These forms of discrimination may be shaped and reinforced by the use of categorical psychiatric diagnostic labels when diagnostic terms become associated with stereotypes of dangerousness, chronicity, unpredictability, or fixed identity categories.

The intervention was grounded in recovery-oriented principles and patient empowerment, avoiding etiological explanations and designed to strengthen three domains: (1) stigma-related knowledge and understanding of mechanisms contributing to discrimination in mental healthcare; (2) behavioral competencies for recognizing and responding to stigmatizing interactions in clinical practice; and (3) attitudes and professional roles supportive of patient advocacy and recovery-oriented care at interpersonal and structural levels. The present study, therefore, aimed to evaluate the feasibility of this pilot intervention and to generate implementation-relevant evidence for integrating anti-stigma training into a psychiatry residency program.

## 2. Materials and Methods

### 2.1. Design and Setting

This study employed a mixed-methods pre–post design. It took place in a psychiatric clinic in Târgu-Mureș, Romania, which delivers psychiatric treatment to people aged over 18 years with any mental disorder. Data collection was conducted between September 2024 and January 2025. The quantitative component allowed for systematic measurement, comparisons and generalizability. Together, the two methods complemented one another: qualitative data enriched an understanding of individual perspectives, while quantitative data enabled objective measurement and broader pattern identification.

### 2.2. Participants

All registered trainees (51 at the time of the study) were invited to participate through a combination of institutional communication (announcement by the academic supervisor) and passive recruitment (posters and informational leaflets). Those who agreed to participate signed the consent form after receiving information about the research, before the start of the first session. Participants were not compensated for attending the training. However, each participant received a certificate of attendance along with 10 continuing medical education credits.

### 2.3. Intervention

The intervention aimed to (i) train mental health professionals to recognize and reduce personal and professional stigma; (ii) address discrimination and self-stigma experienced by patients, and (iii) promote advocacy against interpersonal and structural discrimination. It consisted of four two-hour modules delivered over three weeks. The content of the modules closely followed the manual developed by the READ-MH (Responding to Experienced and Anticipated Discrimination) team while incorporating culturally relevant adaptations. Local adaptations included discussions of context-specific manifestations of structural stigma, such as the underdevelopment of community-based services and institutional policies regarding the use of physical restraints and other corrective measures. Also, given participants’ heightened awareness of stigma among other health professionals [19], greater emphasis was placed on strategies for addressing stigma within interdisciplinary relationships. Several case vignettes were also revised to better reflect stigma-related issues relevant to the Romanian context. Locally available approaches that may support stigma reduction, such as Balint groups, were also highlighted, and specific stigmatizing attitudes were proposed as potential themes for discussion within these groups.

The modules addressed key aspects of mental health-related stigma: its impact on healthcare services, anticipated and experienced discrimination, self-stigma, manifestations of stigma within mental health care, and strategies for its reduction. Each module incorporated interactive lectures and a variety of teaching activities, such as brainstorming sessions, role-playing, group discussions, and case studies. The intervention manual and modules’ content (Supplementary Materials) are publicly available and can be accessed online [33].

### 2.4. Measures

#### 2.4.1. Outcome Measures

Participants were assessed on their knowledge regarding mental health stigma, attitudes toward addressing stigma and discrimination, and behavioral and communication skills. Knowledge was evaluated using a structured questionnaire designed based on the training content [33], ensuring direct alignment between learning objectives and outcome assessment. This approach was selected to capture changes in knowledge that were explicitly targeted by the intervention and to enhance content validity by ensuring that assessed domains reflected the core components delivered during the training. The quiz comprised 6 questions (4 multiple choice and 2 open-ended questions) with a total possible score of 18. The questionnaire covered three key areas: (i) sources of stigma, (ii) manifestations of stigma in mental health care, and (iii) the role of mental health professionals in addressing stigma, both in individuals with mental illness and in their own attitudes and behaviors.

Attitudes were assessed using the Attitudes to Addressing Stigma and Discrimination Scale (ASTAD), developed by members of the INDIGO Network group who developed and evaluated the training for mental health professionals responding to experienced and anticipated discrimination-mental health [23] through adaptation of the Short Alcohol and Alcohol Problems Perception Questionnaire (SAAPPQ) [34]. The SAAPPQ, originally designed to assess health professionals’ attitudes toward working with individuals experiencing alcohol-related issues, demonstrated strong internal consistency and longitudinal invariance. It consists of ten items rated on a 5-point Likert scale (1 = fully agree to 5 = completely disagree), covering five domains: role adequacy, role legitimacy, motivation, work satisfaction, and task-specific self-esteem; and two subscales: role security (role adequacy and legitimacy) and therapeutic commitment (motivation, satisfaction, and self-esteem). To develop the ASTAD, the original items were retained with wording modifications to reflect attitudes toward addressing stigma and discrimination, thereby preserving the SAAPPQ’s psychometric structure while adapting it to the mental health context. In the British validation study, the SAAPPQ showed a good correlation with the extent of postgraduate training in addiction [34].

Behavioral and communication skills were evaluated using an Objective Structured Clinical Examination (OSCE) [35], a standardized and widely used assessment method in medical education. The OSCE scenario reflects common stigma-related interactions in clinical settings. It presented a simulated mental health service user describing both experienced and anticipated discrimination while seeking guidance about disclosing their diagnosis in the context of marriage or employment. Participants were evaluated by the simulated patient who was also involved in delivering the training, across two dimensions: (i) anti-stigma responses: acknowledging the issue, demonstrating empathy, exploring concerns, and supporting informed, value-consistent decision-making; and (ii) stigmatizing behaviors: such as dismissing or doubting reports of discrimination, validating discriminatory acts, attributing avoidance to personal flaws, or giving directive advice on disclosure. The assessment process utilized a standardized marking scheme developed by the INDIGO READ team to enhance reliability and comparability across sites.

#### 2.4.2. Process Measures

We collected attendance records for participants and used a fidelity checklist to assess the implementation of session delivery and participant engagement. Teaching strategies were rated on a three-point scale (0 = not achieved, 1 = partially achieved, 2 = fully achieved) based on selected evidence-based teaching methods. Qualitative data comprising participant feedback gathered at the end of each module, immediately post-intervention and at 3-month post-intervention. The end-of-module feedback questionnaire evaluated participants’ perspectives on the clarity and relevance of the content, session length and delivery methods, achievement of learning objectives, and suggestions for improvement. The overall feedback was obtained at the end of the intervention and at the 3-month follow-up, via individual questionnaires and focus group discussions. The feedback explored participants’ views on the intervention, its impact on their clinical practice, motivation for participation, acceptability, potential barriers, and recommendations for improvement. The quantitative and qualitative assessment instruments used in this study are publicly available and can be accessed online [33].

##### Feasibility

Demand: The implementation of the intervention was assessed in terms of both actual participation and the intention to participate. This was measured by identifying the percentage of the target population that attended at least half of the sessions.

Acceptability: The response of the target population to the intervention was examined to determine its perceived suitability. This aspect focused on whether participants viewed the intervention as relevant and appropriate to their needs. Acceptability was assessed through measures of satisfaction, willingness to continue its use, perceived appropriateness, and observed or reported effects.

Implementation: This component evaluated the effectiveness, quality, and influencing factors of the intervention’s delivery. Qualitative data collected through post-training feedback and 3-month reflections on the training’s perceived impact on professional practice were analyzed using thematic analysis, following a deductive approach guided by predefined domains related to intervention fidelity, perceived relevance, applicability in clinical contexts, barriers to implementation, and facilitators of sustained behavioral change.

Practicality: Participants’ capacity to engage in the intervention activities was assessed using data from the evaluation questionnaire and qualitative feedback.

Adaptability: Any modifications to the original READ-MH guideline that were made to better fit the local context.

Integration: The extent to which the intervention can be integrated into existing workflows or organizational structures. This was assessed qualitatively by interviews and evaluation questionnaires.

Preliminary effectiveness was assessed by measuring changes in participants’ knowledge, attitudes and behavioral and communication skills, complemented by feedback questionnaire responses and focus groups exploring perceived benefits and applicability.

### 2.5. Data Collection and Analysis

The OSCE was administered prior to the knowledge and attitude assessments to minimize potential bias from questionnaire completion. Outcome measures were assessed at baseline, post-training, and at three months after the intervention. Participants completed the knowledge and attitudes questionnaires. Additionally, we collected socio-demographic data: gender, age and academic year.

The 3-month follow-up was selected as a pragmatic timepoint commonly used in educational and training intervention research to assess early sustainability of effects beyond immediate post-intervention change and initial transfer to practice, particularly in studies evaluating skill- and behavior-based outcomes [36].

Baseline outcomes were compared across demographic groups using χ^2^ tests for categorical variables, and Mann–Whitney U tests for continuous variables, in order to determine whether adjustments were required in the analysis. Normality was assessed with the Shapiro–Wilk test. As no significant demographic differences and no deviations from normality were detected, paired t-tests were used to examine within-group changes in outcomes from baseline to post-intervention and to the 3-month follow-up, with Bonferroni correction (adjusted significance threshold *p* < 0.025). Effect sizes were calculated using Cohen’s d to obtain the standardized mean difference (SMD) for continuous variables (knowledge quiz and ASTAD scores), while the Wilcoxon rank biserial correlation (r) was applied for categorical outcomes (OSCE scores). Effect sizes were interpreted as small (SMD = 0.30–0.50), medium (0.50–0.80), or large (>0.80). Statistical analysis was conducted using GraphPad Prism (version 10, GraphPad Software Inc., San Diego, CA, USA). Focus group and individual interview analysis used a deductive approach based on the topic guide [33]. Due to the limited sample size, qualitative data analysis software was not employed; instead, a manual approach was applied.

## 3. Results

Nineteen psychiatry trainees participated in the study, representing approximately 40% of all psychiatry trainees within the hospital. Of these, 80% attended all training sessions, while 20% completed only half of the sessions. The sample included four males and 15 females, reflecting the gender distribution of psychiatrists in the institution. Participants were distributed across training years as follows: first year N = 3 (16%), second year N = 5 (26%), third year N = 5 (26%), and fourth year N = 6 (32%). The mean age of participants was 28.2 years (SD = 1.36).

### 3.1. Knowledge Quiz

Knowledge scores increased by a mean of 6.68 points immediately after training (95% CI 5.86–7.50, *p* < 0.001). This improvement remained evident at follow-up, with a mean difference from baseline of 6.00 points (95% CI 4.98–7.02, *p* < 0.001).

A mean increase of 6.68 points (95% CI 5.86 to 7.50, *p* < 0.001), with a large effect size, was observed immediately after training in the knowledge score. Participants maintained a mean increase of 6.00 points (95% CI 4.98 to 7.02, *p* < 0.001), with a large effect size.

### 3.2. Attitudes to Addressing Stigma and Discrimination Scale (ASTAD)

Participants’ total ASTAD score increased by a mean of 5.00 points (95% CI 3.26–6.74, *p* < 0.001) immediately after training and remained above baseline at follow-up, with a mean difference of 3.01 points, not statistically significant after Bonferroni correction (95% CI 0.26–5.76, *p* ≈ 0.03).

For the role security subscale, scores improved by 2.21 points (95% CI 1.37–3.05, *p* < 0.001) post-intervention, although this gain was not sustained at follow-up.

In contrast, the therapeutic commitment subscale showed both an immediate and persistent improvement, with a 2.79-point (95% CI 1.64–3.94, *p* < 0.001) increase after training and a 2.37-point (95% CI 0.73–4.01, *p* < 0.01) difference from baseline still evident at follow-up (see Table 1).

### 3.3. Objective Structured Clinical Examination (OSCE)

Participants’ overall performance score improved from a mean ordinal score of 1.89 (47.3%) at baseline to 2.84 (71.0%) immediately post-training, representing an increase of approximately 0.95 points (23.7 percentage points; Friedman χ^2^(2) = 26.91, *p* < 0.001). The distribution of OSCE performance categories across time points is presented in Table 2.

### 3.4. Feasibility

Feasibility outcomes were examined to assess the extent to which the intervention could be delivered and engaged within the residency training context. Results are presented across predefined feasibility domains, including demand, implementation, acceptability, practicality, and adaptation. These domains capture different aspects of feasibility, ranging from participation and engagement with the intervention to the experience of delivery and adjustments required during implementation. Findings for each domain are reported in the following subsections.

#### 3.4.1. Demand

The intervention reached approximately 40% of eligible trainees, indicating partial coverage of the target population. Given the voluntary nature of participation, self-selection bias cannot be excluded; however, interview data from participants suggested that the majority did not have prior knowledge of stigma, somewhat mitigating the assumption that participation was driven primarily by pre-existing interest or prior engagement with the topic. Non-participation appeared to be largely explained by structural and contextual factors rather than attitudinal differences. In particular, senior trainees preparing for the national specialty examination did not participate, indicating that competing professional priorities likely played a major role in limiting attendance. The timing of the intervention, scheduled partly during working hours and partly after hours, resulted in scheduling conflicts and inconsistent supervisory support for attendance. While some non-participants cited limited interest, this may have been confounded by workload pressures and limited awareness of the relevance of stigma-focused training. Overall, these factors suggest that reduced participation was driven more by organizational constraints than by systematic attitudinal differences, limiting generalisability.

#### 3.4.2. Implementation

The intervention, originally planned as five 1.5 h modules, was delivered as four 2 h sessions. Four participants (20%) attended only half of the modules; however, all received informational leaflets summarizing the key content. According to the fidelity checklist, all planned components were implemented: each module was completed in full, nearly all presentation materials were used, and both national and local data from the situational analysis were integrated. Role-play exercises were conducted as planned, while live expert-by-experience (EBE) presentations were replaced with a reflective homework task: participants were asked to engage patients in discussions about their experiences of stigma. They were trained to focus on its impact, anticipated and experienced discrimination, self-stigma, and recovery. The homework was followed by group reflection in subsequent sessions. This adaptation was implemented because a local registry and infrastructure for training and involving EBEs were not available at the time of delivery. Although this approach preserved direct contact with individuals with lived experience and opportunities for reflection, it differed from the original READ-MH model by replacing structured contact-based learning led by trained EBEs with participant-mediated clinical encounters. Consequently, intervention fidelity may have been reduced, and comparability with READ-MH outcomes should be interpreted cautiously. The absence of live EBE involvement may also have influenced mechanisms associated with stigma reduction, including perspective-taking, emotional engagement, and challenge of stereotypes, which are considered important active components of contact-based interventions.

Live EBE presentations were not feasible at the time due to the absence of a local registry of trained EBEs.

#### 3.4.3. Practicality

Institutional factors affected participation, with some trainees citing schedule conflicts and limited supervisory support as barriers. Despite endorsement from the education and clinical director to reduce workloads, questionnaire data suggested many participants did not perceive such adjustments or encouragement to attend.

#### 3.4.4. Adaptation

We modified the intervention by replacing live EBE testimonials with direct trainee–patient discussions followed by structured group reflection.

#### 3.4.5. Integration

The findings indicated that participants perceived the intervention as suitable for integration into psychiatric training curriculum during working hours and recommended that it be delivered over an extended timeframe to avoid substantial interference with clinical duties.

All participants recommended implementing the intervention within the host organization. Reported hindrances included limited support from institutional leadership, lack of interest from the mental health professionals, and excessive workload demands. The intervention demonstrated a strong potential for expansion. Guidelines and training materials were reported to be clear, well-structured, and easily adaptable, facilitating replication in different settings.

#### 3.4.6. Acceptability and Effectiveness

All participants completed pre and post-intervention outcome measures, satisfactory questionnaires and participated either in a focus group or individual interview. Post-intervention and follow-up assessments indicated a reduction across all scales and subscales. For those outcomes with statistically significant pre–post changes, effect sizes were in the medium to large range. All participants reported they were satisfied with the course; the information was clear, highly relevant for clinical practice, the objectives were achieved entirely, the format of presentation was beyond expectations for all participants, and they expressed their appreciation for the practical aspects of the course. Follow-up interviews indicated the trainees applied the knowledge and skills acquired during the training and reported increased confidence in their communication and engagement with patients. They further expressed their beliefs that these changes positively influence patients’ self-perception and how they cope with experienced or anticipated discrimination. Participants reported several benefits from the training: recognition of negative attitudes, prejudices, and manifestations of stigma, as well as an increased awareness that psychiatrists themselves can be a source of stigmatization. They also noted increased sensitivity to self-stigma and other stigma-related experiences in patients, the importance of seeing the patient beyond the diagnosis, and the psychiatrist’s role in shaping patients’ self-perception and their responses to situations of discrimination. The following elements of the training were identified as the most efficient: first, the interactive theoretical component helped increase awareness of stigmatizing aspects in their clinical practice. They reported being more aware before the training of stigma patterns among other healthcare professionals than among psychiatrists themselves. Second, role-playing exercises, case studies, and facilitated group discussions were perceived as having the greatest impact on developing communication skills and enhancing confidence in their ability to bring about change.

## 4. Discussion

The intervention led to improvements in stigma-related knowledge, attitudes, and behavioral competencies, with knowledge gains being the most stable over time, suggesting that the training content was conceptually clear and effectively supported the retention and internalization of theoretical concepts underpinning attitudinal and behavioral change. Attitudinal improvements were reflected in an overall increase in the ASTAD score immediately after the intervention, suggesting that the training enhanced participants’ perceived ability and legitimacy in addressing stigma, as well as their motivation and confidence in this role. However, at follow-up, only the therapeutic commitment subscale remained significantly improved, while role security (comprising perceived adequacy and legitimacy) returned toward baseline. This pattern may indicate that although the training temporarily increased participants’ sense of preparedness and legitimacy in addressing stigma, these gains were less sustained over time without continued reinforcement. By contrast, the sustained improvement in therapeutic commitment, which reflects motivation, work satisfaction, and task-specific self-esteem, suggests a more robust effect on the underlying willingness and confidence to engage with patients experiencing stigma.

The qualitative findings provide further insight into this pattern. Participants identified experiential learning methods, such as role-playing, case-based discussions, and group reflection, as the most valuable training components, contributing to greater self-efficacy and motivation to address stigma. These activities likely underpinned the sustained improvement in therapeutic commitment. By contrast, behavioral components associated with role adequacy and legitimacy appear more dependent on ongoing institutional reinforcement and opportunities for real-world application. These observations are consistent with prior evidence indicating that attitudinal components related to intrinsic motivation and self-perception are more stable than those linked to perceived knowledge or role definition, which typically require continuous reinforcement to maintain [37,38,39].

The attitudinal changes were only partially reflected in behavior change. Although participants demonstrated improvements in communication and stigma-responsive skills after training, performance gains shifted from “harmful or ineffective” to “somewhat effective,” but did not reach the “highly effective” level. This pattern is consistent with implementation and the training literature showing that sustained changes in clinical practice typically require ongoing training and integration into routine rather than a single training exposure [40,41,42,43]. World Health Organization’s workplace training guidelines reported small positive effects or no effects observed on behaviors at follow-up (3–6 months) for many programs [44]. A representative meta-analysis of Potthoff et al. concludes that habitual behaviours are strong determinants of clinical practice and that deliberate reinforcement and repeated practice are needed to break old habits and form new ones [40]. While increased therapeutic commitment may create the motivation to act differently, translating this motivation into consistently applied skills likely depends on opportunities for sustained experiential learning and feedback in real clinical contexts. Long-term impact is therefore likely contingent on both individual-level engagement and organizational commitment to maintaining stigma-responsive care as a core professional competency.

The intervention was generally well accepted and perceived as relevant to clinical practice, enhancing participants’ confidence in addressing mental health-related stigma. Engagement was moderate, with 40% of eligible trainees participating, comparable to participation rates reported in other anti-stigma training studies for mental health professionals, which range from 22 to 86% [15,17,45]. The predominance of female participants reflected the workforce composition of the host institution, while the absence of final-year trainees likely reflected scheduling constraints rather than a lack of interest.

These findings collectively suggest that the intervention was both feasible and acceptable within the hospital setting, even amid academic and clinical demands. Comparison of feasibility data with the existing literature was limited, as only one of the few studies evaluating anti-stigma interventions for mental health professionals reported feasibility-related information, which was similar to our findings [17].

However, several barriers to expansion were identified. These include high workload demands that may limit staff availability, potential lack of support from institutional leadership, and challenges related to the implementation process, particularly the requirement for the intervention to be delivered within existing working hours. Addressing these factors will be essential to ensure the sustainability and scalability of the intervention.

Furthermore, there is a documented demand for the intervention beyond the pilot group, with potential to target additional categories of mental health professionals. Interviews conducted within the host organization indicated that psychiatric nurses were willing to engage in future training, recognizing the intervention’s relevance and importance to their professional practice.

Implication for research and practice:

The current findings emphasize the importance of designing stigma-reduction initiatives that extend beyond short-term educational interventions. The absence of structured stigma-related training within residency curricula represents a systemic gap observed not only in Romania but across many LMIC settings. Integrating anti-stigma principles into formal training curricula, supervision structures, and routine clinical education, rather than offering isolated workshops, may strengthen role security over time, support earlier development of reflective and patient-centered professional identities, and encourage interdisciplinary collaboration.

Periodic reinforcement mechanisms may be particularly important for maintaining gains over time and reducing skill decay in behavioral competencies that require repeated practice and feedback. Integration of contact-based approaches and reflective learning opportunities within clinical training environments may represent feasible pathways for promoting sustained changes in attitudes and practice.

Limitations:

Several limitations should be considered when interpreting these findings. First, the modest sample size and single-site design limit generalizability to other residency programs and healthcare settings. Second, participation was voluntary, introducing the possibility of self-selection bias, whereby participants with greater pre-existing interest in mental health stigma may have been more likely to enroll.

Third, the absence of a control group limits causal inference regarding the intervention’s effectiveness. The relatively short follow-up period further constrains conclusions regarding the sustainability of observed changes over time. Additionally, behavioral outcomes were assessed through simulated interactions rather than direct observation of routine clinical encounters, which may not fully reflect the transfer of acquired skills into clinical practice.

Finally, adaptations introduced during implementation, including the replacement of live expert-by-experience involvement with reflective homework activities, may have influenced intervention fidelity and should be considered when comparing findings with prior implementations of the training model.

Future Directions:

Future research should prioritize more rigorous evaluation designs, including randomized controlled trials with comparison groups and larger multisite samples. Studies comparing alternative implementation formats (e.g., workshop-only delivery versus longitudinal integration within residency curricula) would provide evidence regarding optimal intervention intensity, duration, and scalability.

Extended follow-up assessments, inclusion of behavioral observation in clinical settings, and incorporation of institutional-level outcomes may provide stronger evidence regarding long-term effectiveness. Comparative evaluations of reinforcement strategies, such as peer mentoring, reflective supervision, and digital follow-up modules, may help identify sustainable and cost-effective implementation pathways.

Future studies should also strengthen the involvement of experts by experience in both intervention development and delivery. Contact-based education co-delivered by trained service users has consistently been associated with improvements in empathy, attitudinal change, and understanding of the lived experience of stigma. Establishing local networks of trained service users may therefore represent a sustainable and contextually relevant model for future educational initiatives.

## 5. Conclusions

The intervention proved to be feasible and well accepted within the clinical setting, being perceived as relevant to professional practice and contributing to increased participants’ confidence in addressing mental health-related stigma.

Quantitative findings demonstrated improvements in knowledge, attitudes, and behavioral competencies, with varying degrees of sustainability. Knowledge gains were the most robust and stable, suggesting that the theoretical component of the training was effective. Attitudinal changes were only partially maintained over time, with sustained improvement in therapeutic commitment, while perceived role adequacy and legitimacy declined toward baseline. Behavioral improvements were observed but remained limited.

Qualitative findings highlighted the key role of experiential learning methods in enhancing self-efficacy and motivation. However, they also suggest that the translation of these gains into consistent clinical practice requires repeated exposure, feedback, and integration into real-world contexts. A single training intervention appears insufficient to produce lasting behavioral change.

Long-term impact is likely dependent on both individual engagement and organizational support. Identified barriers, including high workload, time constraints, and limited institutional support, must be addressed to ensure sustainability and scalability.

Overall, the study underscores the need to integrate anti-stigma training into residency curricula and supervision structures through longitudinal approaches and continuous reinforcement strategies. Future research, including randomized controlled trials and long-term evaluations, is necessary to determine the most effective models for sustaining attitudinal and behavioral change.

## Figures and Tables

**Table 1:** Descriptive statistics and pairwise comparisons for knowledge and attitudes scores.

	Timeline	Mean (SD)	Pairwise Comparison	T	*p*	Cohen’s d	Effect Size
Knowledge quiz	Before	13.79 (1.13)					
	After	20.47 (1.17)	Before vs. after	17.12	<0.001	5.8	Large
	Follow-up	19.79 (1.78)	Before vs. follow-up	12.4	<0.001	4.02	Large
ASTAD total score	Before	38.47 (2.86)					
	After	43.47 (2.76)	Before vs. after	6.04	<0.001	1.77	Large
	Follow-up	41.48 (5.1)	Before vs. follow-up	2.3	0.03	0.72	Moderate
Role security	Before	16.47 (1.65)					
	After	18.68 (0.95)	Before vs. after	5.5	<0.001	1.64	Large
	Follow-up	17.11 (2.33)	Before vs. follow-up	0.98	0.3	0.31	-
Therapeutic commitment	Before	22 (1.8)					
	After	24.79 (2.32)	Before vs. after	5.12	<0.001	1.34	Large
	Follow-up	24.37 (2.93)	Before vs. follow-up	3.04	<0.01	0.7	Moderate

SD = Standard deviation. Effect size interpretation (Cohen’s d): small = 0.2–0.49, moderate = 0.5–0.79, large ≥ 0.8. After Bonferroni correction, *p* < 0.03 was considered statistically significant.

**Table 2:** Distribution of OSCE ratings pre/post-intervention.

OSCE	Before n (%)	After n (%)
Harmful	6 (31.6%)	0 (0%)
Ineffective	9 (47.4%)	3 (15.8%)
Somewhat effective	4 (21%)	16 (84.2%)
Highly effective	0 (0%)	0 (0%)
Mean ordinal performance score (95% CI)	1.89 (1.54 to 2.25)	2.84 (2.66 to 3.02)
Score improvement (95% CI)		+0.95 points (0.62 to 1.28)
Friedman χ^2^ (df)		26.91 (2)
*p* value		<0.001

## Data Availability

Anonymized individual-level data, derived outcome scores, and analytic materials underlying the findings of this study will be made available upon reasonable request to the corresponding author, subject to institutional approval and applicable ethical and data protection requirements.

## Supplementary Materials

The intervention materials (the training manual, presentation slides, and assessment measures) are available for download at: https://indigo-group.org/resources/intervention-development-and-implementation/ (accessed on 29 June 2026).

## Abbreviations

The following abbreviations are used in this manuscript:

LMICs	Low and Middle-Income Countries
HICs	High-Income Countries
ASTAD	Addressing Stigma and Discrimination
SAAPPQ	Short Alcohol and Alcohol Problems Perception Questionnaire
OSCE	Objective Structured Clinical Examination
READ	Responding to Experienced and Anticipated Discrimination
EBE	Expert By Experience

## References

[1] Henderson C., Stuart H., Hansson L. (2016). Lessons from the results of three national antistigma programmes. Acta Psychiatr. Scand..

[2] Thornicroft G., Mehta N., Clement S., Evans-Lacko S., Doherty M., Rose D., Henderson C. (2016). Evidence for effective interventions to reduce mental health-related stigma and discrimination. Lancet.

[3] Hansson L., Stjernswärd S., Svensson B. (2016). Changes in attitudes, intended behaviour, and mental health literacy in the Swedish population 2009–2014: An evaluation of a national antistigma programme. Acta Psychiatr. Scand..

[4] Thornicroft C., Wyllie A., Thornicroft G., Mehta N. (2014). Impact of the “Like Minds, Like Mine” anti-stigma and discrimination campaign in New Zealand on anticipated and experienced discrimination. Aust. N. Z. J. Psychiatry.

[5] Raj T.C. (2022). The effectiveness of mental health disorder stigma-reducing interventions in the healthcare setting: An integrative review. Arch. Psychiatr. Nurs..

[6] Gronholm P.C., Henderson C., Deb T., Thornicroft G. (2017). Interventions to reduce discrimination and stigma: The state of the art. Soc. Psychiatry Psychiatr. Epidemiol..

[7] Henderson C., Noblett J., Parke H., Clement S., Caffrey A., Gale-Grant O., Schulze B., Druss B., Thornicroft G. (2014). Mental health-related stigma in health care and mental health-care settings. Lancet Psychiatry.

[8] Zaske H., Freimuller L., Wolwer W., Gaebel W. (2014). Anti-stigma competence for mental health professionals: Results of a pilot study of a further education programme for people working in psychiatric and psychosocial settings. Fortschr. Neurol. Psychiatr..

[9] Li J., Fan Y., Zhong H.Q., Duan X.L., Chen W., Evans-Lacko S., Thornicroft G. (2019). Effectiveness of an anti-stigma training on improving attitudes and decreasing discrimination towards people with mental disorders among care assistant workers in Guangzhou China. Int. J. Ment. Health Syst..

[10] Giralt Palou R., Prat Vigué G., Tort-Nasarre G. (2020). Attitudes and stigma toward mental health in nursing students: A systematic review. Perspect. Psychiatr. Care.

[11] Heim E., Henderson C., Kohrt B.A., Koschorke M., Milenova M., Thornicroft G. (2020). Reducing mental health-related stigma among medical and nursing students in low-and middle-income countries: A systematic review. Epidemiol. Psychiatr. Sci..

[12] Deb T., Lempp H., Bakolis I., Vince T., Waugh W., Henderson C. (2019). Responding to experienced and anticipated discrimination (READ): Anti -stigma training for medical students towards patients with mental illness—Study protocol for an international multisite non-randomised controlled study. BMC Med. Educ..

[13] Sreeram A., Cross W.M., Townsin L. (2022). Anti-stigma initiatives for mental health professionals-A systematic literature review. J. Psychiatr. Ment. Health Nurs..

[14] Gupta S., Kumar A., Kathiresan P., Pakhre A., Pal A., Singh V. (2024). Mental health stigma and its relationship with mental health professionals: A narrative review and practice implications. Indian J. Psychiatry.

[15] Bayar M.R., Poyraz B.C., Aksoy-Poyraz C., Arikan M.K. (2009). Reducing mental illness stigma in mental health professionals using a web-based approach. Isr. J. Psychiatry Relat. Sci..

[16] Lagunes-Cordoba E., Alcala-Lozano R., Lagunes-Cordoba R., Fresan-Orellana A., Jarrett M., Gonzalez-Olvera J., Thornicroft G., Henderson C. (2022). Evaluation of an anti-stigma intervention for Mexican psychiatric trainees. Pilot Feasibility Stud..

[17] Henderson C. (2016). Qualitative analysis of mental health service users’ reported experiences of discrimination. Acta Psychiatr. Scand..

[18] Lagunes-Cordoba E., Davalos A., Fresan-Orellana A., Jarrett M., Gonzalez-Olvera J., Thornicroft G., Henderson C. (2021). Mental health service users’ perceptions of stigma, from the general population and from mental health professionals in Mexico: A qualitative study. Community Ment. Health J..

[19] Manescu E.A., Henderson C., Paroiu C.R., Boaca A.C., Marchean H., Mihai A. (2025). Stigma Toward People With Mental Illness Among Romanian Psychiatry Trainees: A Mixed-Methods Study. Cureus.

[20] Schulze B. (2007). Stigma and mental health professionals: A review of the evidence on an intricate relationship. Int. Rev. Psychiatry.

[21] Knaak S., Mantler E., Szeto A. (2017). Mental illness-related stigma in healthcare: Barriers to access and care and evidence-based solutions. Healthc. Manag. Forum.

[22] Wang K., Link B.G., Corrigan P.W., Davidson L., Flanagan E. (2018). Perceived provider stigma as a predictor of mental health service users’ internalized stigma and disempowerment. Psychiatry Res..

[23] Henderson C., Ouali U., Bakolis I., Berbeche N., Bhattarai K., Brohan E., Cherian A., Girma E., Gronholm P.C., Gurung D. (2022). Training for mental health professionals in responding to experienced and anticipated mental health-related discrimination (READ-MH): Protocol for an international multisite feasibility study. Pilot Feasibility Stud..

[24] Guerrero Z., Iruretagoyena B., Parry S., Henderson C. (2024). Anti-stigma advocacy for health professionals: A systematic review. J. Ment. Health.

[25] Potts L.C., Bakolis I., Deb T., Lempp H., Vince T., Benbow Y., Waugh W., Kim S., Raza S., Henderson C. (2022). Anti-stigma training and positive changes in mental illness stigma outcomes in medical students in ten countries: A mediation analysis on pathways via empathy development and anxiety reduction. Soc. Psychiatry Psychiatr. Epidemiol..

[26] Griffith J.L., Kohrt B.A. (2016). Managing stigma effectively: What social psychology and social neuroscience can teach us. Acad. Psychiatry.

[27] Cozman D., Nemeș B., Buciuta A., Dima C., Stoean R. (2021). Inpatient psychiatric services use during the COVID-19 pandemic in Romania. Medichub.

[28] Manescu E.A., Henderson C., Paroiu C.R., Mihai A. (2023). Mental health related stigma in Romania: Systematic review and narrative synthesis. BMC Psychiatry.

[29] Ministry of Health of Romania, Ministry of Education Ordin No. 1141/1386/2007 Regarding Residency Training Programs in Romania. https://legislatie.just.ro/Public/DetaliiDocument/86760.

[30] Angermeyer M.C., Holzinger A., Carta M.G., Schomerus G. (2011). Biogenetic explanations and public acceptance of mental illness: Systematic review of population studies. Br. J. Psychiatry.

[31] Carter L., Read J., Pyle M., Morrison A.P. (2017). The Impact of Causal Explanations on Outcome in People Experiencing Psychosis: A Systematic Review. Clin. Psychol. Psychother..

[32] Thornicroft G., Brohan E., Rose D., Sartorius N., Leese M. (2009). Global pattern of experienced and anticipated discrimination against people with schizophrenia: A cross-sectional survey. Lancet.

[33] Materials from INDIGO Partnership Project. https://indigo-group.org/resources/intervention-development-and-implementation/.

[34] Anderson P., Clement S. (1987). The AAPPQ revisited: The measurement of general practitioners’ attitudes to alcohol problems. Br. J. Addict..

[35] Khan K.Z., Ramachandran S., Gaunt K., Pushkar P. (2013). The Objective Structured Clinical Examination (OSCE): AMEE Guide No. 81. Part I: An historical and theoretical perspective. Med. Teach..

[36] Mehler M., Balint E., Gralla M., Pößnecker T., Gast M., Hölzer M., Kösters M., Gündel H. (2024). Training emotional competencies at the workplace: A systematic review and metaanalysis. BMC Psychol..

[37] Zhamaliyeva L., Ablakimova N., Batyrova A., Veklenko G., Grjibovski A.M., Kudaibergenova S., Seksenbayev N. (2025). Interventions to Reduce Mental Health Stigma Among Health Care Professionals in Primary Health Care: A Systematic Review and Meta-Analysis. Int. J. Environ. Res. Public Health.

[38] Albarracin D., Shavitt S. (2018). Attitudes and Attitude Change. Annu. Rev. Psychol..

[39] Kohrt B.A., Jordans M.J.D., Turner E.L., Sikkema K.J., Luitel N.P., Rai S., Singla D.R., Lamichhane J., Lund C., Patel V. (2018). Reducing stigma among healthcare providers to improve mental health services (RESHAPE): Protocol for a pilot cluster randomized controlled trial of a stigma reduction intervention for training primary healthcare workers in Nepal. Pilot Feasibility Stud..

[40] Potthoff S., Rasul O., Sniehotta F., Marques M., Beyer F., Thomson R., Presseau J. (2019). The relationship between habit and healthcare professional behaviour in clinical practice: A systematic review and meta-analysis. Health Psychol. Rev..

[41] Shelton R.C., Cooper B.R., Stirman S.W. (2018). The Sustainability of Evidence-Based Interventions and Practices in Public Health and Health Care. Annu. Rev. Public Health.

[42] Sullivan A., Elshenawy S., Ades A., Sawyer T. (2019). Acquiring and Maintaining Technical Skills Using Simulation: Initial, Maintenance, Booster, and Refresher Training. Cureus.

[43] Nevedal A.L., Widerquist M.A.O., Reardon C.M., Arasim M., Jackson G.L., White B., Burns M., Fix G.M., DeLaughter K., Cutrona S.L. (2024). Understanding pathways from implementation to sustainment: A longitudinal, mixed methods analysis of promising practices implemented in the Veterans Health Administration. Implement. Sci..

[44] WHO (2022). Guidelines on Mental Health at Work.

[45] Ng Y.P., Rashid A., O’Brien F. (2017). Determining the effectiveness of a video-based contact intervention in improving attitudes of Penang primary care nurses towards people with mental illness. PLoS ONE.

